# Health research priority setting in selected high income countries: a narrative review of methods used and recommendations for future practice

**DOI:** 10.1186/1478-7547-12-23

**Published:** 2014-11-18

**Authors:** Jamie Bryant, Rob Sanson-Fisher, Justin Walsh, Jessica Stewart

**Affiliations:** Priority Research Centre for Health Behaviour, School of Medicine & Public Health, University of Newcastle, Callaghan, NSW 2308 Australia; Hunter Medical Research Institute, New Lambton Heights, NSW Australia; School of Medicine and Public Health, University of Newcastle, Callaghan, NSW Australia

**Keywords:** Review, Health, Research priorities

## Abstract

Research priority setting aims to gain consensus about areas where research effort will have wide benefits to society. While general principles for setting health research priorities have been suggested, there has been no critical review of the different approaches used. This review aims to: (i) examine methods, models and frameworks used to set health research priorities; (ii) identify barriers and facilitators to priority setting processes; and (iii) determine the outcomes of priority setting processes in relation to their objectives and impact on policy and practice.

Medline, Cochrane, and PsycINFO databases were searched for relevant peer-reviewed studies published from 1990 to March 2012. A review of grey literature was also conducted. Priority setting exercises that aimed to develop population health and health services research priorities conducted in Australia, New Zealand, North America, Europe and the UK were included. Two authors extracted data from identified studies.

Eleven diverse priority setting exercises across a range of health areas were identified. Strategies including calls for submission, stakeholder surveys, questionnaires, interviews, workshops, focus groups, roundtables, the Nominal Group and Delphi technique were used to generate research priorities. Nine priority setting exercises used a core steering or advisory group to oversee and supervise the priority setting process. None of the models conducted a systematic assessment of the outcomes of the priority setting processes, or assessed the impact of the generated priorities on policy or practice. A number of barriers and facilitators to undertaking research priority setting were identified.

The methods used to undertake research priority setting should be selected based upon the context of the priority setting process and time and resource constraints. Ideally, priority setting should be overseen by a multi-disciplinary advisory group, involve a broad representation of stakeholders, utilise objective and clearly defined criteria for generating priorities, and be evaluated.

## Review

### Introduction

The primary aim of research priority setting is to gain consensus about areas where increased research effort including collaboration, coordination and investment will have wide benefits to society. Priority-driven research has a clearly defined purpose, with an emphasis on answering questions of key importance that are likely to have a significant impact on knowledge or practice in the short to medium term [1]. The use of a systematic, explicit and transparent process of setting health research priorities ensures that research is funded that has the greatest potential public health benefit, that research funding and outputs are aligned with the needs of decision makers [2], and that there is efficient and equitable use of limited resources, with less duplication of research effort [3]. Priority setting should be as evidence-based as possible, while also incorporating the views of a wide range of stakeholders [4].

A wide range of approaches for setting research priorities exist. While there is no consensus about a gold standard or best practice model, general principles for setting health research priorities have been suggested [5–8]. In 2010, Viergever and colleagues published a nine item checklist for priority setting providing recommendations about processes that should be considered before, during and after undertaking priority setting [6]. However to date, there has been no comprehensive review of health research priority setting in high income countries, the barriers and facilitators of different approaches, nor the effectiveness of different models of priority setting in terms of outcomes. This information is needed to provide guidance to research and policy makers of high income countries who wish to undertake priority setting exercises.

The aim of this review is to:

1. Examine methods, models and frameworks used to set health research priorities;
2. Identify barriers and facilitators to priority setting processes; and
3. Determine the outcomes of priority setting processes in relation to their objectives, and impact on policy or practice.

## Methods

### Literature search

Medline, Cochrane, and PsycINFO databases were searched for relevant peer-reviewed studies published from 1990 to March 2012. A combination of MeSH and keywords were used (see Table 1). Each identified article or report was examined by two reviewers (JB and JW) to assess relevance. The reference lists of relevant articles were also reviewed to identify further potentially relevant sources of information. Google and Google Scholar searches were also conducted to search for additional grey literature, including government and organisational reports of priority setting processes, with the first one hundred results from each search reviewed. The tables of contents of the following five relevant journals were manually searched between 2007 and February 2012: Cost Effectiveness and Resource Allocation; Health Research Policy and Systems; Journal of Public Health Practice and Management; International Journal for Equity in Health; and Health Policy.

**Table 1:** **Search terms**

Database	Search terms
**PsycINFO**	[Setting priorities (title/abstract) *OR* priority setting (title/abstract) *OR* Resource allocation (MeSH)]; *AND* [Research (title/abstract) *OR* Experimentation (MeSH)]; limit published 1990-current.
**Medline**	Health Priorities (MeSH) *OR* priority setting [title/abstract] *OR* Resource allocation (MeSH); *AND* research (MeSH); limit published 1990-current.
**Cochrane**	Health priorities (title/abstract) *OR* priority setting (title/abstract) *AND* research.
**Google and google scholar (first 100 results)**	Establishing health research priorities; Research priority setting framework; Setting research priorities; National Institute Health priority setting; National Health Service priority setting.

### Inclusion and exclusion criteria

Any priority setting exercise that aimed to develop research priorities for population health and health services programs in Australia, New Zealand, North America, Europe and the UK were included. The content areas for health research were not restricted. The range of research methodologies included within frameworks was broadly defined and could include: intervention research, implementation and translation research, comparative effectiveness research, quality improvement research, and other forms of rigorous program evaluation. Only priority setting frameworks which were actually implemented were included. Priority setting processes carried out in non-health related fields, or approaches that were implemented to set health care or service delivery priorities were excluded. The final priority setting processes included in the review were selected to ensure that a diverse range of priority setting approaches were considered within the scope of this narrative review.

### Data extraction

Authors JB and JW extracted data from included priority setting exercises. Data included: the aim of the priority setting process; the scope of the process in terms of the specificity of developed priorities; the methods and criteria used to generate priorities; the methods and criteria used to rank priorities; the stakeholders included in the priority setting process; and the success of the model in meeting its stated objectives.

## Results

### Search outcomes

**Figure 1:**
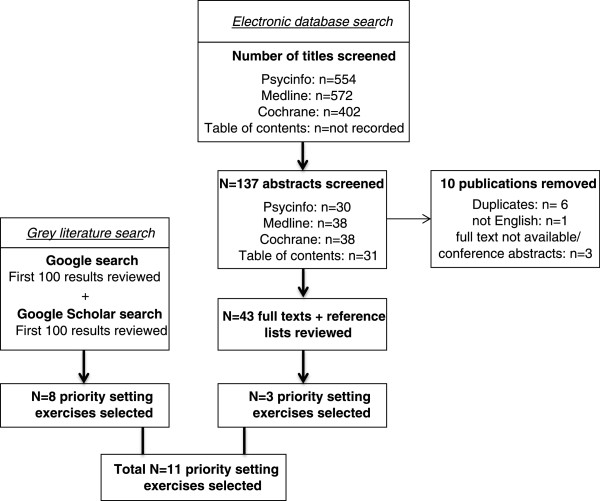
**Flowchart of search strategy.**

Figure 1 shows the outcomes of the implemented search strategy.

### Identified models

Eleven diverse priority setting exercises carried out in Australia [9–12], the United Kingdom [13–18], the United States [19] and Canada [20–23] were identified (see Table 2). Three priority setting exercises related specifically to Indigenous health [9–11, 23], two related to primary and secondary care research [14, 15, 17], and one each related to cancer [12], anaesthesiology [13], asthma [16], general health [20–22], service delivery research [18] and comparative effectiveness research [19].

**Table 2:** **Summary of identified priority setting processes (N = 11)**

Reference	Aim of priority setting process scope country	Method used to generate priorities	Criteria used to guide generation of priorities	Criteria or method for ranking priorities	Stakeholders represented	Success of model in meeting objectives
***National Health and Medical Research Council (NHMRC); NHMRC Road Map I and II*** [9] **,** [10]	Support and foster research to improve the health of Aboriginal and Torres Strait Islander people.	*Road Map I:*	*Road Map I:* Six research themes were established by a working group and research questions were elicited within these themes: (i) Patterns of risk, disease and death; (ii) Resilience and wellness; (iii) Health service research; (iv) Health impact of non-health sector policies and programs; (v) Previously under-researched populations and communities; (vi) Improving research capacity	*Road Map I:* Priorities not ranked.	- Peak Advisory Bodies for Aboriginal Health	Not reported
		i. Broad themes identified by advisory group				
					- Health Organisations	
					- Researchers	
	Broad themes and specific research questions.					
					- Aboriginal and Torres Strait Islander community representatives	
		ii. Call for written comments from stakeholders				
	Australia.					
		iii. Series of workshops held to refine research issues and themes.				
***Cooperative Research Centre for Aboriginal Health (CRCAH); Setting and meeting priorities in Indigenous health research*** [11]	Develop a model for collaborative development of research projects.	Roundtable discussion convened with industry partners and researchers.	Existing priorities of the research program used.	Priorities ranked by board according to perception of greatest impact. Both social merit and scientific merit considered.	Representatives from:	Not reported
					- Aboriginal Health	
					- Relevant government agencies	
					- Health care funders	
					- Peak bodies	
					- Interested researchers	
					- Community leaders	
	Specific research questions developed from pre-determined themes.					
	Australia.					
***Social Sciences and Humanities Research Council (SSHRC), Canada; Establishing priorities for Aboriginal Research in Canada*** [23]	Develop Aboriginal research as a priority area.	National call for briefs on shaping a proposed Aboriginal health research Agenda.	Call for submissions in five areas: (i) Program priorities; (ii) Ethical guidelines; (iii) Methodologies; (iv) Decision-making; (v) Building capacity – nurturing indigenous scholarship.	Priorities not ranked.	Representatives from:	Not reported
					- Aboriginal organisations	
					- Academic organisations	
	Specific research questions.					
					- Government	
					- Community organisations	
	Canada.					
***Institute of Medicine (IOM); Developing National Priorities for Comparative Effectiveness Research*** [19]	Determine research priorities for comparative effectiveness research.	i. Stakeholder input sought via email and letter correspondence	None reported. Respondents were invited to identify three priority areas and (i) provide data to justify each choice; (ii) assign each topic to a single primary research area; and (iii) identify the study population and identify a proposed methodology.	Four *portfolio criteria* were used by the committee to ensure a balance of questions reflecting a wide range of research areas, methodologies, populations. *Condition level criteria* and *Priority topic-level criteria* were then used to rank priorities.	- Media	Not reported
					- Policy makers	
					- Academics	
					- Consumers	
		ii. Web questionnaire circulated to more than 20,000 individuals seeking specific priority research recommendations			- Researchers	
	Specific research questions.				- Health care industry	
					- Health care providers	
	United States.					
					- Staff of government agencies	
		iii. Public session held for stakeholder presentations.				
***Canadian Health Services Research Foundation, in partnership; Listening for Direction I, II, III*** [20] **-** [22]	Identify areas where research investment is most likely to improve system-level decision making.	i. Environmental scan of policy issues	Stakeholders given timeframe to consider research outcomes: (i) Listening for Direction I – Medium term (2–5 years); (ii) Listening for Direction II – Short term (6–24 months) and medium term (2–5 years); (iii) Listening for Direction III – Short term (6–24 months) and long term (3–10 years).	i. Translation and sorting sessions used to identify emergent themes from earlier stages.	- Research funders;	Not clear
					- Decision makers (hospital and health region managers, clinical leaders)	
		ii. Decision making groups, research groups and funding organisations surveyed				
					- Researchers	
					- Research users (consultants, professional associations, knowledge brokers)	
	Broad themes and illustrative research questions.					
				ii. Themes categorised as *primary* or *secondary* according to how frequently they were encountered.		
				iii. A single top priority emerged as the most frequently encountered. Otherwise priorities were not ranked further.		
		iii. Workshops held to discuss priority issues				
	Canada.					
***National Health Service (NHS); Setting Priorities for Research in Primary and Secondary Care*** [14] **,** [15]	Identify and prioritise needs for research and development in primary and secondary health care.	i. Advisory group established	(i) *Need:* likely benefit of research to NHS and patient care; relevance to policy initiatives; burden of disease; costs to the service and to patients; and level of practice variation;	Priorities ranked using the same criteria used to generate priorities. Members of advisory panel scored topics on a five point scale according to criteria with priorities ranked	- Nurses	Not reported
		ii. Two researchers asked to provide the advisory group with a critical overview of current evidence				
					- Clinicians (generalist and specialist)	
					- Management (purchasers and providers)	
					- Research	
	Broad research topics.				- Consumers	
	United Kingdom.	iii. Three separate panels convened to review evidence provided and seek stakeholder input using a variety of methods.	(ii) *Research and development potential*: feasibility of research including availability of existing methodology and resources; likelihood of research being implemented; management commitment to the issue; study design; and participants.	according to mean scores.		
***National Coordinating Centre for Service Delivery and Organisation, National Health Service (NHS); Establishing Research Priorities for Service Delivery Research- National Listening Exercise*** [18]	Set research priories for service delivery research by the NHS in England.	i. Expert forum convened to advise on composition of focus groups and issues that should be addressed	Participants asked to generate priorities that could be achieved within next 3–5 years.	Priorities not ranked.	- Consumers	Not reported
					- Educators	
		ii. 22 focus groups with stakeholders held				
					- Research funders	
					- Innovators	
		iii. Findings from focus groups validated against other sources of information				
					- Researchers	
		iv. Priorities translated into research themes				
	Research themes.					
	United Kingdom.					
***Consulting about Priorities for the NHMRC National Breast Centre*** [12]	Identify agreed areas of priority for the work of the newly established National Breast Centre.	Representative group of stakeholders invited to attend a workshop hosted by state based cancer organisations. Attendees generated a list of priorities prior to the meeting and presented them to the group. Less structured workshops held with Aboriginal and other groups.	No explicit criteria. Participants drew on personal experiences and perspectives.	Nominal group technique. Priorities ranked based on discussion and group consensus.	- Women diagnosed with or at heightened risk of breast cancer and their partners;	Outcome of process was used to draft the NHMRC National Breast Cancer Centre’s strategic direction document.
					- Health professionals (medical oncology, radiation oncology, pathology, providers of Breast Screen Australia, nurses)	
	Areas of priority.					
	Australia.					
					- Public health experts	
					- Administrators	
***National Institute of Academic Anaesthesia Research Priority Setting Exercise*** [13]	Establish priorities for anaesthesia and perioperative medicine and to direct the attention of researchers and funding bodies to these priorities.	i. List of research questions generated via completion of a questionnaire by anaesthesiologists and lay representatives.	Respondents asked to generate research questions that could ‘lead to improvements in patient care, patient safety and patient outcomes’. No other criteria stated. Patients drew on own experiences and perspectives.	Respondents scored presented priorities according to importance on 10 point likert scale rather than ranking them against each other.	- Anaesthesiologists	Not reported
					- Lay representatives of a patient liaison group	
		ii. Results collated into theme areas to produce a list for further prioritisation.				
	Specific research questions.					
	United Kingdom.					
		Second questionnaire sent asking anaesthesiologists and lay representatives to identify their level of support for each identified area. A brief vignette, one to two pages in length, was prepared for each question in the second survey and provided to the expert panel.				
**James Lind Alliance; Identifying and prioritizing uncertainties: Patient and clinician agreement in identification of research questions** [16]	Develop a prioritized ranking of treatment uncertainties in asthma that require further research.	i. Collaboration between organisations established	No criteria provided.	Three rounds of a nominal group technique. Participants at prioritization workshop were first asked to rank the list of 21 treatment uncertainties presented in order of importance prior to workshop. Nominal group process then occurred until consensus achieved.	- Asthma patients (Asthma UK staff and patient advocates)	Not reported
					- Researchers	
					- Clinical specialists	
		ii. Explicit statements of research need identified from clinical guidelines, reviews and research recommendations				
		iii. Patient survey developed and sent to consumers and placed on public website				
	Specific research questions.					
	United Kingdom.					
***London Region Research and Development Programme; Using consensus methods to establish multidisciplinary perspectives on research priorities for primary care*** [17]	To examine the feasibility of using consensus techniques to determine priority research questions on the effectiveness, cost and quality of prescribing.	Nominal group interview with 12 participants. Participants asked “*What research questions on the effectiveness, cost and quality of prescribing should be given priority for support as research questions in this locality?”*	None.	Scores and items from priority generation stage reviewed by steering group and six priority themes developed. Stratified sampling used to recruit balanced sample of pharmacists, general practitioners and nurses who engaged in a two-round postal Delphi process.	- Pharmacists	Not reported
					- General Practitioners	
					- Nurses	
	Broad themes that were turned into specific research questions.					
	United Kingdom.					

### Level of priority setting and specificity of developed priorities

Nine of the eleven reviewed priority setting exercises aimed to set health research priorities at a national level [9, 10, 12–16, 18, 20–23]. One priority setting exercise aimed to generate local priorities [17], and one aimed to establish priorities to guide decision-making about developing and funding specific projects [11]. There were differences in the specificity of the priorities that each model aimed to generate. Three of the eleven reviewed models identifying only broad research themes [12, 14, 15, 18] while eight models identified specific research questions [9–11, 13, 16, 17, 19–23]. Of those identifying specific questions, several used a staged process, first deciding on broad priority areas, then generating specific research questions [9, 10, 20–22].

### Methods for generating priorities

A diverse range of strategies including calls for submission, stakeholder surveys and questionnaires, stakeholder interviews, workshops, focus groups, roundtables, the Nominal Group technique and the Delphi technique were used to generate research priorities. Each of these strategies are outlined below and the advantages and disadvantages of each are outlined in Table 3.

**Table 3:** **Advantages and disadvantages of different methods of generating priorities**

	Advantages	Disadvantages
**Calls for submission**	• Enable a wide range of stakeholders to be reached.	• Requires stakeholders to have a level of written expertise in order to respond.
	• Inexpensive and non-resource intensive for the commissioning organisation.	
**Stakeholder questionnaires/ surveys**	• Potential to reach a large number and wide range of stakeholders.	• Challenges with designing surveys that are appropriate for stakeholders of various backgrounds/expertise.
		• Interpretation may be required to collate responses if open-ended questions asked.
**Workshops, focus groups or roundtables**	• Increases the likelihood that different views can be openly debated.	• Some individuals may have greater dominance in a group situation leading to views or concerns of individuals being neglected.
**Nominal group technique**	• Facilitates equal participation of all group members.	• Structured process can minimise discussion and reduce opportunities for the development and refinement of ideas.
	• Reduces the domination of the discussion by a single person or group of people.	
	• Results in a set of prioritised solutions or recommendations that are agreed to democratically by the majority of group members.	
**Delphi technique**	• Does not require face-to-face meetings and therefore is relatively free of social pressure, dominance of individuals or groups, and is inexpensive [24].	• Numerous rounds of questionnaires can be time consuming and requires commitment from individuals over a period of time.
		• Vulnerable to differential response rates and can have high rates of attrition between rounds [17].
		• May force a middle-of-the-road consensus, militating independent judgements [25].
**Public input session**	• Promotes public awareness of the topic areas being addressed.	• Public setting may inhibit expression of ideas which could draw criticism or debate.
	• Allows for a wide range of stakeholders to contribute.	
		• Public setting may disadvantage/discourage non-expert stakeholders from contributing alongside experts.
		• Practical/time constraints in receiving input from large numbers of participants.

#### Workshops, focus groups or roundtables

The majority of approaches used workshops [14, 15], roundtables [23], focus groups [12, 18], or approaches that included focus groups, workshops or round tables in combination with other approaches [9–12, 20–22] to bring key stakeholders together to generate research priorities. Each differed in the format used. Most merged representatives from multiple stakeholder groups together [9, 10, 14, 15, 23], while two used a combination of merged and separate stakeholder groups. For example, in the priority setting exercise carried out by the National Health and Medical Research Council (NHMRC) Breast Centre to identify agreed areas of priority [12], merged stakeholder focus groups were conducted in each state in Australia, followed by three separate focus groups with Aboriginal and Torres Strait Islander people, people from non-English speaking backgrounds, and people from rural and remote areas. The use of special workshops was considered to provide unique insights, raising issues not identified in representative workshops [12]. The priority setting exercise to set research priories for service delivery research by the National Health Service (NHS) in England also used a combination of approaches. Sixteen mixed groups were held across health regions, with an additional six focus groups held comprising experts in specific groups only; one group was held specifically with consumers, one group with educators, one group with research funders, one group with innovators and two groups with researchers [18].

#### Stakeholder surveys or questionnaires

Stakeholder surveys were used to generate priority areas in four of the included priority setting exercises [13, 16, 19–22]. Responses were typically collated, refined and arranged into categories or themes which become the basis for further discussion or prioritisation. The size and scope of the surveys and questionnaires differed. In the Institute of Medicine (IOM) priority setting exercise [19], a web-based questionnaire was sent to more than 20,000 individuals on the institutes’ database as well as the media, academics, policy makers, researchers, physicians, health care providers, federal government agencies and individuals and organisations interested in health policy. A total of 1,758 responses were received.

#### Calls for submission or comment

Calls for submission or comment seek to utilise stakeholders’ personal and/or professional perspectives and expertise to generate priority areas. Two priority setting exercises carried out to establish Indigenous research priorities sought submissions from the public. The Canadian Social Science and Humanities Research Council exercise put out a national call for briefs as the first step of the priority setting process [23]. Five criteria developed by a steering committee were provided to shape the direction of responses from participants. The Australian NHMRC process used both a call for submissions and a series of workshops to generate priorities [9, 10]. Calls for submissions were sought within six research themes pre-determined by a Research Advisory Working Group.

#### Nominal group technique

The nominal group technique is a structured group information gathering process that aims to combine idea generation and consensus building into a single meeting [26]. A question is posed, then responses from participants are sought, collated, and disseminated to the wider group. Participants are then asked to prioritize the ideas put forward by group members. The nominal group technique was used to generate priorities in two priority setting processes [12, 17]. The London Region Research and Development Programme process to set priorities on the effectiveness, cost and quality of prescribing in primary care [17] used a structured nominal group interview in which individual participants presented ideas for research questions to a group. This process provided an opportunity for discussion and helped to establish a common representation of research questions from apparently divergent ideas. In the NHMRC Breast Cancer Centre priority setting exercise [12], representative stakeholders were asked to generate a list of priorities from an individual perspective prior to a group meeting. The structured nominal groups process were reported to result in reasonably high levels of agreement about priorities across the workshops held.

#### Delphi technique

The Delphi technique is a structured model undertaken predominantly using questionnaires. Participants answer a questionnaire, then the results (usually a statistical representation of the group response including reasons for judgements [25]) are circulated to all participants. Participants are encouraged to revise their original responses in light of the responses of other participants, allowing sharing of information and reasoning among participants. Generally two or more rounds are conducted, with the answers of participants converging towards consensus. The Delphi technique was used in the London Region Research and Development Programme priority setting exercise carried out to set priorities on the effectiveness, cost and quality of prescribing in primary care [17]. A two-round postal Delphi process was undertaken with pharmacists, general practitioners and nurses. Low response rates were obtained in both the round one (53%) and round two (38%) questionnaires.

#### Public input session

The IOM exercise to set research priorities for comparative effectiveness research also utilised a public meeting to seek input from stakeholders [19]. Fifty-four experts were invited to address the committee, making 3 minute long presentations as well as written statements which were made publicly available on the IOM website.

### Ranking generated priorities

Prioritisation is a process whereby individuals or groups place in rank order identified research priorities in terms of their importance or significance. Specific criteria are normally provided to aid this process. Seven of the eleven working examples used a variety of processes to rank priorities (see Table 3). Techniques included subjective ranking based on perception of social and scientific merit [11], simple counting of the number of times a priority area was mentioned with the most frequently mentioned ranked first [20–22], ranking based on sophisticated criteria (including data on prevalence, mortality, morbidity, cost and variability, utility of area for decision making, information gaps, variability in care, and gaps in translation [19]) and ranking using a five point scales designed to capture the need for the research (likely benefit of research to the organisation and patient care; relevance to policy initiatives; burden of disease; costs to the service and to patients; and practice variation) and research potential (feasibility, degree of management commitment to the issue; study design; and participants) [14, 15]. Three use structured processes of the nominal group technique [12, 16] and the Delphi technique [17] to rank priorities.

### Advisory group oversight and stakeholder involvement

Nine of the eleven priority setting exercises reported the use of a core steering or advisory group to oversee and supervise the priority setting process [9–11, 13–19, 23]. The composition, level of involvement and function of each steering or advisory group varied. The most common function of the core steering or advisory groups was synthesising, refining and or translating into themes priority areas generated by stakeholders [14, 16–18, 23]. Other roles of the core steering or advisory group included: developing criteria to guide the generation of priority areas by stakeholders [23]; ranking developed priorities [11]; generating criteria to guide ranking of priorities [19]; agreeing on a set of common terms and/or definitions to be used during the priority setting process [16, 19]; and determining which stakeholders should be consulted [18].

Stakeholder input was a feature of all eleven reviewed priority setting processes. However, the breadth of stakeholder involvement and the way stakeholders were involved differed. Some priority setting approaches conducted separate consultation processes with individual groups of stakeholders, while others identified a broad range of stakeholders and brought people from each one of these groups together. Nine of the eleven reviewed models sought consumer input. Six of these models used consultative consumer participation [9, 10, 12, 13, 18, 19, 23] and three used collaborative approaches [11, 14–16] while none used a consumer controlled approach.

### Outcome evaluation

None of the models identified conducted a systematic assessment of the outcomes of the priority setting processes, or assessed whether the generated priorities had any impact on policy or practice [3]. There was very little evidence of the reliability and validity of priorities generated through priority setting frameworks, and no evaluation or reflection on whether the generated research priorities resulted in improvements to important outcomes. Three approaches did however assess whether identified priorities reflected participants perceptions of discussions by carrying out validation surveys [12, 18, 20–22] all with positive results. One additional exercise assessed participant satisfaction with the process [12].

### Identified barriers to carrying out research priority setting

A number of barriers to conducting priority setting exercises were identified.

#### Multi-component methods are resource intensive

Each of the priority setting exercises utilised a multi-component approach, which required considerable time and resources. In some instances the level of demand placed on resources was initially underestimated or later criticised. The James Lind Alliance encountered difficulties conducting the asthma exercise due to constraints on clinician and patient time, which prolonged the process to approximately six months [16]. The Cooperative Research Centre for Aboriginal Health also received criticism that the facilitated development approach was too time-consuming and resource-intensive, requiring the involvement and co-ordination of many participants across multiple stages [11].

#### Difficulties conceptualising the process and intended outcomes and generating initial priorities

In several priority setting exercises, there was difficulty among both experts and non- experts in conceptualising some aspects of the process. In the James Lind Alliance exercise which involved setting priorities for asthma research, patients involved in the priority setting process had difficulties conceptualising research opportunities which were referred to as treatment ‘uncertainties’ [16]. Despite repeated clarification, many of the patient responses had to be removed as they did not address the area of research interest. The NHS priority setting exercise also found problems with the advisory group struggling to clearly define the area of research and distinguish the interface between primary and secondary care [14, 15]. In the National Institute of Academic Anaesthesia exercise [13], individuals found the process of generating research ideas burdensome and challenging, which contributed to a relatively low response rate from participants. Lay members in particular reported difficulties in forming specific research questions as required.

#### Difficulties making decisions within the advisory group

In the NHS priority setting exercise, there was a perception of a lack of background knowledge of the current state of the literature within the advisory group, which made it difficult for the group to make decisions [14, 15]. The advisory group also struggled with distinguishing between the importance of research and its feasibility.

#### Ideas not being suggested by researchers out of fear that idea would be appropriated by others

Priority setting exercises may be inhibited by the traditionally competitive nature of research. In the National Institute of Academic Anaesthesia exercise, there was a perception that some anaesthesiologists did not submit research ideas out of concern their idea would be appropriated by others [13].

### Identified facilitators to conducting research priority setting

#### Structured techniques were perceived as useful

Three priority setting processes used the nominal group method to generate and/or decide on priorities [12, 16, 17]. In all three instances, the structured approach was perceived as useful in facilitating agreement about priorities. Giving participants the opportunity to discuss the rationale for presented ideas was thought to help build group cohesion, facilitate the refinement of disparate ideas [17], and assist in reaching consensus [16].

#### Piloting of questionnaire

Prior to distributing a questionnaire to determine national priorities for anaesthesia and perioperative medicine, the National Institute for Academic Anaesthesia piloted the questionnaire with 32 respondents from two teaching hospitals [13]. This proved useful as the initial questionnaire, which asked respondents to suggest a research question and the primary outcome measure and patient group, was considered ‘too complex and demanding’. As a result, the questionnaire was altered to ask respondents to suggest research topics only in more general terms. The priorities were then developed into specific research questions by the advisory group.

#### Separate consultation exercises for ‘non-professional’ consumers or special groups

In two instances, the use of separate consultation exercises for consumers or special groups has been found to be valuable. In setting priorities for the NHMRC Breast Centre, special workshops were held to consult with Aboriginal and Torres Strait Islander women, women of non-English speaking background, and women living in rural or remote areas [12]. These workshops used a less structured method of gaining consensus than other workshops, but were considered important to consider the needs of women who had more difficulty accessing information or services. After analysis of priorities identified by each of the workshops held, the special workshops were considered to provide unique insights, and raised issues not identified in other workshops. Similarly, in the NHS exercise, a separate consumer consultation exercise, in the form of a focus group of informal carers, was convened as one attempt to sample the views of ordinary users as opposed to the “professional” consumers who responded to the formal consultation exercise [14, 15]. This input was helpful to the advisory group as a prompt to consider the needs of individual users when setting priorities.

## Discussion

This review shows a failure to evaluate priority setting processes and a lack of consensus about appropriate priority setting methodologies [6]. It is therefore not possible to provide strong evidence-based recommendations about optimum methods to set research priorities. Consequently, any attempts to develop a priority setting process must rely on the critical appraisal of the existing literature, expert consensus, and the relevance and necessities of the local socio-political and policy environment. The following recommendations are made:

### A multi-disciplinary advisory group should oversee the priority setting process

A well-managed and resourced multi-disciplinary advisory group should oversee the priority setting exercise. Such an advisory group provides credibility to the process of determining research priorities, and ensures the developed priorities are relevant and feasible. The group should elect a member to chair the group. A process should be put in place to manage any potential conflicts of interest.

### Broad representation of stakeholders is critical

Involvement of a broad representation of stakeholders was seen in the majority of priority setting models as a strength of the process, consistent with notions that such inclusion provides credibility and transparency to the process, and ensures that developed priorities are relevant, feasible, and meet actual health care needs [27–29]. Involvement of researchers may mean they are more likely to commit to undertaking research within identified priority areas [30], and involvement of policy makers may mean a greater likelihood of knowledge transfer and implementation of research outcomes [6]. The guiding principle in selecting stakeholder groups for consultation should be one of inclusivity [31]. Depending upon the technicality of the selected approach, it may be necessary to provide additional support to consumers members of the workgroup through individual sessions outside working group meetings to clarify objectives and ensure members are comfortable with what is being asked of them [32].

### Objective, clearly defined criteria should guide the generation of priorities

Clear and specific criteria for eliciting and ranking potential priorities should be determined by an advisory group before seeking stakeholder input. Whether specific research questions or broad priority areas are generated as an outcome of the priority setting process, these should be determined based on the purpose and context of the priority setting process [6, 7].

### The impact of the priority setting processes should be evaluated

An outcome evaluation should be integrated into the priority setting process to provide evidence for future priority setting processes. Although difficult to undertake, as has been suggested elsewhere [33], an outcome evaluation could include tracking and reporting of acceptability and perceived usefulness of individuals involved in the process; the number and type of new initiatives generated that relate to the priority areas in defined time period after implementation; the number and type of research projects undertaken related to each endorsed research question; the key outputs of any initiatives funded as a direct or indirect result of the priority setting process; and the outcomes associated with each one of the research initiatives, including indices such as number of people trained in research methodology, number of publications, number of publications by designated priority determined area, the amount of measurement, descriptive and intervention research, the impact of the research in informing policy, program or health service delivery design.

### Strengths and limitations of this review

This narrative review examined priority setting processes carried out in selected high income countries with similar healthcare systems. Only priority setting processes actually implemented were examined to ensure the feasibility, acceptability and barriers to carrying out the priority setting activity could be examined. While systematic methods were used to identify relevant priority setting processes, the scope of this review was limited to a diverse but relatively small sample of priority setting processes, and is not intended to be exhaustive. While we aimed to determine the outcomes of priority setting processes in relation to their objectives and impact on policy or practice**,** we only assessed the impacts as reported in the examined publication, rather than conducting an additional broad and comprehensive literature review. Therefore it is possible that some broad policy and practice implications have been missed. Additionally, the review focuses on priority setting exercises carried out only in selected high income countries and so should be considered within this context only.

## Conclusions

There is a no consensus about a gold standard or best practice model for health research prioritization [6]. Nevertheless, the recommendations arising from this review are consistent with other guidance literature on this topic [5–8, 33]. The method used should be selected based upon the context of the priority setting process, as well as time and resource constraints. Priority setting should be overseen by a multi-disciplinary advisory group, should involve a broad representation of stakeholders, should utilise objective and clearly defined criteria for generating priorities and should be evaluated.
